# Myeloid‐Derived Suppressor Cells: Immunoregulatory Roles and Therapeutic Prospects in Immune‐Mediated Hematological Disorders

**DOI:** 10.1155/jimr/5577070

**Published:** 2026-01-31

**Authors:** Qi Liu, Kunpeng Zhang, Shun He, Xinru Jia, Jingjing Liu, Dijiong Wu, Baodong Ye

**Affiliations:** ^1^ Department of Hematology, The First Affiliated Hospital of Zhejiang Chinese Medical University (Zhejiang Provincial Hospital of Chinese Medicine), Hangzhou, Zhejiang, China, zjhtcm.com; ^2^ The First School of Clinical Medicine, Zhejiang Chinese Medical University, Hangzhou, Zhejiang, China, zcmu.edu.cn; ^3^ School of Pharmaceutical Sciences, Zhejiang Chinese Medical University, Hangzhou, Zhejiang, China, zcmu.edu.cn; ^4^ National Traditional Chinese Medicine Clinical Research Base (Hematology), Hangzhou, Zhejiang, China; ^5^ Department of Oncology and Hematology, Wenzhou Hospital of Integrated Traditional Chinese and Western Medicine Affiliated to Zhejiang Chinese Medicine University, Wenzhou, Zhejiang, China

**Keywords:** immune-mediated hematological diseases, immunosuppression, myeloid-derived suppressor cells

## Abstract

Myeloid‐derived suppressor cells (MDSCs) are a heterogeneous population of immature myeloid cells that have been increasingly defined and characterized over the past two decades, known for their remarkable capacity to expand and exert immunosuppressive effects in various pathological contexts, such as malignancies, infections, and inflammatory diseases. Although MDSCs have attracted significant attention in tumor immunology, their multifaceted roles in immune‐mediated hematological disorders remain comparatively understudied and less understood, especially in contrast to their well‐documented functions in both solid and hematological malignancies. This review delves into the biological characteristics and functional mechanisms of MDSCs, as well as their dual immunomodulatory roles in various immune‐mediated hematological disorders such as immune thrombocytopenia and aplastic anemia. Substantial evidence indicates that in these diseases, the expansion, recruitment, and function of MDSCs are significantly altered, with their levels closely correlated to disease activity. Based on these findings, we further explore the therapeutic potential of targeting MDSCs. Manipulating MDSCs offers a pioneering perspective for the development of next‐generation immunotherapies, which holds promise for reshaping the current treatment landscape of immune‐mediated hematological disorders.

## 1. Introduction

Myeloid‐derived suppressor cells (MDSCs) are a heterogeneous population of immature cells derived from myeloid hematopoietic progenitor cells, characterized by potent immunosuppressive functions. In recent years, the role of MDSCs in malignant tumors has been extensively investigated, with compelling evidence demonstrating their pro‐tumorigenic roles within the tumor microenvironment (TME) [1]. Studies have revealed that elevated levels of circulating MDSCs are strongly associated with poor prognosis and reduced survival rates in patients with solid malignancies [2]. Furthermore, increased MDSC abundance significantly diminishes therapeutic sensitivity to chemotherapy, radiotherapy, and immunotherapy in both hematological malignancies (e.g., Hodgkin lymphoma [HL], multiple myeloma [MM]) and solid tumors [3–6]. Although the involvement of MDSCs in solid and hematological malignancies has garnered considerable attention, their roles in immune‐mediated hematological disorders remain relatively underexplored. For instance, the precise mechanisms through which MDSCs operate in conditions such as immune thrombocytopenia (ITP), aplastic anemia (AA), graft‐versus‐host disease (GVHD), and chronic idiopathic neutropenia (CIN) remain poorly characterized. This review therefore aims to systematically delineate the origin, surface markers, immunosuppressive functions, and potential roles of MDSCs in immune‐mediated hematological disorders, with the goal of providing novel insights into immunoregulatory mechanisms underlying these diseases.

## 2. The Historical Tracing of MDSCs

The discovery and research trajectory of MDSCs can be traced back to the early 20^th^ century, with early observations of aberrant myeloid differentiation in hematopoietic malignancies [7]. By the 1970s, “natural suppressor cells”—myeloid cells lacking lymphocyte markers but suppressing T cells—were identified [8]. In the 1990s, advancements in animal models revealed a myeloid‐derived suppressor subpopulation co‐expressing Gr‐1 and CD11b in tumor‐bearing mice, while functionally analogous CD34^+^CD14^+^ cell clusters were identified in human TMEs, marking the initial phenotypic characterization of MDSCs [8–10]. However, due to the marked heterogeneity of these cells, early literature employed inconsistent nomenclature, with terms such as “immature myeloid cells”, “myeloid suppressor cells”, and “Gr‐1^+^ myeloid cells” appearing across studies [11–14]. A pivotal resolution was achieved in 2007 when the international scientific community formally adopted the term “myeloid‐derived suppressor cells (MDSCs)”, which integrated their bone marrow origin, immunosuppressive functionality, and pathological expansion dynamics, thereby resolving longstanding terminological controversies [15]. Nevertheless, the phenotypic complexity (e.g., granulocytic vs. monocytic subsets), morphological diversity, and functional plasticity of MDSCs continued to contribute to heterogeneous research outcomes. To address these challenges, Bronte’s team established systematic identification criteria in 2016, emphasizing the need for multimodal characterization that integrates phenotypic markers (CD11b^+^Gr‐1^+^ in mice or CD11b^+^CD33^+^HLA‐DR^−/low^ in humans), molecular signatures such as the expression of immunosuppressive mediators (e.g., arginase‐1 [Arg‐1] and reactive oxygen species [ROS]), and functional validation through demonstrated suppression of T‐cell activity—a framework that has since provided a critical foundation for standardized MDSC research [16]. While classically defined as immunosuppressive in tumors, MDSCs display context‐dependent plasticity. In psoriasis, for example, they lose suppression, promote IL‐17 via RORγt/NFAT1, and exert pro‐inflammatory effects. This functional adjustment across pathologies has prompted the proposal of the term “myeloid‐derived adjuster cells” (MDACs) [17].

## 3. Deciphering MDSCs: Ontogeny, Functional Heterogeneity, and Bifaceted Immunoregulatory Mechanisms in Tumor and Inflammatory Disease Pathogenesis

### 3.1. Ontogeny of MDSCs

Current consensus posits that MDSCs fundamentally represent progenitor or precursor cells released from the bone marrow or spleen [18, 19]. From a developmental biology perspective, myeloid cells mature through a hierarchical differentiation cascade originating from hematopoietic stem cells (HSCs), progressing through multipotent progenitor(MPP) cell, common myeloid progenitors (CMPs), and granulocyte‐monocyte progenitors (GMPs). Recent advances have delineated the granulocytic lineage trajectory, wherein proneutrophils—the earliest committed progenitors—proliferate and differentiate into late‐stage precursors termed preneutrophils, ultimately generating immature and mature neutrophil subsets. In the monocytic lineage, GMPs differentiate into common monocyte progenitor (cMoP), which transition through a transient premonocyte (TpMo) stage before maturing into Ly6C^high^ monocyte [20]. Inflammatory conditions, especially in the TME, can prematurely release immature myeloid cells into circulation, increasing systemic heterogeneity. This is termed “emergency myelopoiesis” [21]. An alternative hypothesis suggests that the TME may reprogram the phenotype or metabolism of neutrophils, monocytes, or their progenitors, thereby conferring MDSC‐like characteristics [22].

### 3.2. Expansion and Activation of MDSCs

Recent advances highlight the “two‐signal model” for MDSC regulation, involving a proliferation/expansion pathway and a functional activation pathway [19]. The proliferation pathway is driven by cytokines, such as granulocyte‐macrophage colony‐stimulating factor (GM‐CSF), macrophage colony‐stimulating factor (M‐CSF), interleukin‐6 (IL‐6), and vascular endothelial growth factor (VEGF) from the TME or bone marrow stromal cells [23]. These bioactive molecules drive the expansion of MDSCs by activating the signal transducer and activator of transcription 3 (STAT3)–interferon regulatory factor 8 (IRF8)–CCAAT/enhancer‐binding protein β (C/EBPβ) signaling axis and synergistically regulating key molecules including RB1, Notch, adenosine A2B receptor (A2BR), and NOD‐like receptor family pyrin domain‐containing 3 (NLRP3) inflammasome [24, 25]. The activation pathway is orchestrated by pro‐inflammatory mediators secreted from tumor stroma or activated T cells, including interferon‐γ (IFN‐γ), IL‐1β, IL‐4/IL‐13, S100 calcium‐binding protein A8/A9 (S100A8/A9), high mobility group box 1 (HMGB1), cyclooxygenase‐2 (COX2), and prostaglandin E2 (PGE2). Mechanistically, the signaling pathways driving MDSC activation involve STAT1, STAT6, and nuclear factor kappa‐light‐chain‐enhancer of activated B‐cells (NF‐κB) [24, 25]. In inflammatory contexts like GVHD or systemic lupus erythematosus (SLE), MDSC activation may paradoxically promote pro‐inflammatory responses [26–28].

Beyond the aforementioned growth factors and pro‐inflammatory mediators, studies have revealed that tumor cell‐derived exosomes significantly promote MDSCs formation by delivering bioactive molecules such as migration‐inhibition factor (MIF) and ectonucleotide pyrophosphatase/phosphodiesterase 1 (ENPP1) [29]. Concurrently, free microRNAs (miRNAs) in the TME and exosome‐carried miRNAs (e.g., miR‐9, miR‐21, miR‐155) inhibit MDSCs apoptosis and enhance their expansion through targeted suppression of tumor suppressor genes (e.g., PTEN, suppressor of cytokine signaling 1 [SOCS1]) [30] and myeloid differentiation‐related genes such as runt‐related transcription factor 1 (RUNX1) [31–33]. These networks synergize with cytokine pathways to amplify MDSC development [34, 35].

**Figure 1:**
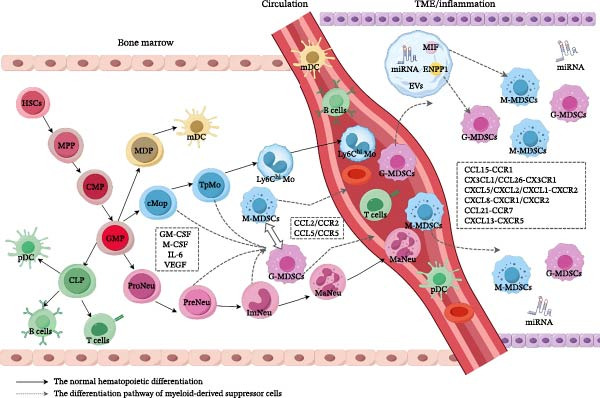
Differentiation of MDSCs. Under physiological conditions, hematopoietic stem cells (HSCs) hierarchically mature through multipotent progenitors (MPPs), common myeloid progenitors (CMPs), and granulocyte‐monocyte progenitors (GMPs), which bifurcate into granulocytic and monocytic lineages. In the granulocytic pathway, GMPs generate proneutrophils (ProNeu) that proliferate and mature into preneutrophils (PreNeu), ultimately differentiating into immature (ImNeu) and mature neutrophil (MaNeu) subsets. In the monocytic lineage, GMPs differentiate into common monocyte progenitors (cMoPs), which transiently transition through premonocytes (TpMo) before maturing into Ly6C^high^ monocytes (Ly6C^high^Mo). Under pathological conditions, GM‐CSF, M‐CSF, IL‐6, and VEGF drive robust expansion of immature myeloid cells. These cells are mobilized into peripheral circulation via the CCL2/CCR2 and CCL5/CCR5 chemokine axes, followed by CXCL‐mediated recruitment to tumor microenvironments (TMEs) or inflammatory niches, where, under the synergistic action of extracellular vesicles (EVs) and cell‐free miRNAs, they undergo further expansion and functional maturation of myeloid‐derived suppressor cells (MDSCs). MIF: migration‐inhibition factor; ENPP1: ectonucleotide pyrophosphatase/phosphodiesterase 1; CLP: common lymphoid progenitor; MDP: macrophage‐dendritic cell progenitor; mDC: myeloid dendritic cell; pDC: plasmacytoid dendritic cell. This figure was originally created by Figdraw (www.figdraw.com).

Based on the dynamic features of cellular migration, Karin et al. [36] proposed an innovative “four‐phase event model” to elucidate the generation mechanism of MDSCs. The process begins with myelopoiesis in the bone marrow or secondary lymphoid organs (Phase I), followed by mobilization into peripheral circulation driven by axes such as CCL2/CCR2 and CCL5/CCR5 (Phase II). Subsequent tumor homing is coordinately regulated by multiple chemokine signaling pathways, including CCL15‐CCR1, CX3CL1/CCL26‐CX3CR1, CXCL5/CXCL2/CXCL1‐CXCR2, CXCL8‐CXCR1/CXCR2, CCL21‐CCR7, and CXCL13‐CXCR5 [37]. Finally, the moder proposes a tumor retention phase where specific chemokine receptors and adhesion molecules are potentially involved [38]; however, its precise regulatory mechanisms remain a theoretical hypothesis requiring experimental validation (Phase IV) (Figure 1).

### 3.3. Heterogeneity and Phenotypic Characterization of MDSCs: From Subset Classification to Mature Neutrophil Reprogramming

Based on morphological and functional characteristics, MDSCs are classified into two major subsets: granulocytic myeloid‐derived suppressor cells (G‐MDSCs) or polymorphonuclear myeloid‐derived suppressor cells (PMN‐MDSCs), and monocytic myeloid‐derived suppressor cells (M‐MDSCs), which phenotypically and morphologically resemble neutrophils and monocytes, respectively [39]. In mice, MDSCs are identified as CD11b^+^Gr‐1^+^ cells, subdivided into two CD11b^+^Ly6G^-^Ly6C^high^ M‐MDSCs and CD11b^+^Ly6C^low^Ly6G^+^ PMN‐MDSCs. In humans, MDSCs are generally defined as CD11b^+^CD33^+^HLA‐DR^-/low^ cells, with PMN‐MDSCs as CD14^-^CD15^+^ and M‐MDSCs as CD14^+^CD15^-^ [40]. Alternatively, PMN‐MDSCs have also been characterized as CD14^-^CD15^+^CD66b^+^CD16^+^CD11b^+^CD33^+^HLA‐DR^-^ [41]. Specific markers like lectin‐type oxidized LDL receptor 1 (LOX‐1) and fatty acid transport protein 2 (FATP2) have been identified as associated with the immunosuppressive function of PMN‐MDSCs in cancer [42, 43], though their role in non‐cancer contexts remains unclear. Additional human subsets include early‐MDSCs (Lin^-^CD33^+^HLA‐DR^-^) [44, 45] and fibrocytic MDSCs [46, 47].

Although the conventional view posits that MDSCs with immunosuppressive functions primarily reside in an immature differentiation state and are typically enriched in the peripheral blood mononuclear cell (PBMC) layer following density gradient centrifugation [22, 48], emerging studies have revealed that mature neutrophils in the peripheral blood (PB) of head and neck cancer and urological cancer patients, as well as in the bone marrow of multiple myeloma patients, exhibit significant immunosuppressive properties [49, 50]. Notably, Pettinella et al. demonstrated that mature normal‐density neutrophils CD66b^+^CD10^+^CD16^+^ (mNDNs) and mature low‐density neutrophils (mLDNs) isolated from colony‐stimulating factor (G‐CSF)‐administered healthy donors not only potently suppress T‐cell proliferation and NK cell‐mediated tumor cytotoxicity but also share identical molecular phenotypes with mature PMN‐MDSCs found in the PB of non‐small cell lung cancer and head and neck cancer patients. These cells specifically co‐express CD52, CD84, and prostaglandin E receptor 2 (PTGER2), suggesting that mature PMN‐MDSCs undergo unique, differentiation‐associated reprogramming during their development [51].

### 3.4. Dual Immunoregulatory Roles of MDSCs: Mechanisms of Immune Suppression, Pro‐Inflammatory Effect, and Cross‐Talk With Adaptive and Innate Immune Subsets

#### 3.4.1. T Cells

MDSCs coordinately suppress T‐cell activity through multidimensional metabolic reprogramming, driving immune evasion and disease progression. By overexpressing Arg‐1 and inducible nitric oxide synthase (iNOS), MDSCs competitively deplete L‐arginine in the TME, leading to impaired synthesis of the T‐cell receptor (TCR) ξ‐chain and subsequent suppression of T‐cell proliferation and functional dysregulation [52, 53]. Concurrently, MDSCs secrete indoleamine 2,3‐dioxygenase 1 (IDO1) to catalyze the degradation of tryptophan into kynurenine. Mechanistically, IDO1 catalyzes the rate‐limiting step in this conversion, generating L‐kynurenine. This metabolite has been demonstrated to interact with the aryl hydrocarbon receptor (AHR), which not only inhibits CD8^+^ T‐cell proliferation and induces apoptosis but also promotes the expansion of regulatory T cells (Tregs), thereby reshaping an immunosuppressive microenvironment and contributing to the mitigation of GVHD [54–56].

Furthermore, lipid metabolic reprogramming enhances the immunosuppressive capacity of MDSCs: PMN‐MDSCs upregulate FATP2 to facilitate arachidonic acid uptake and synthesize prostaglandin E2 (PGE2) via a COX2‐dependent pathway, amplifying immunosuppressive effects [43], while sustained lipid uptake and fatty acid oxidation (FAO) provide continuous energy to maintain T‐cell suppression [57, 58]. Additionally, MDSC metabolism generates ROS, including superoxide (O2−), hydrogen peroxide (H_2_O_2_), and peroxynitrite (ONOO−) [59], which contribute to DNA damage and nitration of critical proteins such as TCR, CD3, and CD8, further impairing T‐cell functionality [60]. In immune‐mediated hematological diseases, reduced frequency or impaired function of MDSCs directly leads to insufficient suppression of autoreactive T cells or cytotoxic T lymphocytes (CTLs), constituting a key event in disease pathogenesis. Conversely, restoring or expanding MDSCs through interventions such as post‐transplant cyclophosphamide (PTCy) or cyclosporine A (CsA) can effectively inhibit alloreactive T cells and ameliorate disease severity [61, 62]. Beyond metabolic mechanisms, MDSCs also secrete immunosuppressive cytokines like TGF‐β and IL‐10 [63]. Notably, studies have revealed that M‐MDSCs expressing programed death‐ligand 1 (PD‐L1) directly eliminate CD8^+^ T cells in vitro, highlighting their role in mediating T‐cell depletion [64].

#### 3.4.2. NK Cells

Multiple studies utilizing murine models and clinical tumor patients have demonstrated a significant negative correlation between NK cells and MDSCs within the TME. MDSCs suppress NK cell infiltration and functions through multiple pathways [65, 66]. Key mechanisms include downregulating the expression of critical activating receptors on NK cells, such as NKG2D (natural killer group 2 member D), natural cytotoxicity receptors (NCRs, e.g., NKp30, NKp44, NKp46), and their signal‐transducing subunit CD247, thereby impairing NK cell recognition of tumor cells [67, 68]. Additionally, MDSCs directly inhibit NK cell activity via secretion of immunosuppressive factors, including TGF‐β, IL‐10, ROS, Arg‐1, and iNOS [66]. For instance, in head and neck tumor models, splenic accumulation of CXCR2^+^MDSCs markedly reduced NK cell functionality [67]. Clinically, co‐culture of peripheral blood‐derived PMN‐MDSCs from primary or metastatic lung cancer patients with NK cells significantly suppressed NK cell production of pro‐inflammatory cytokines (e.g., IFN‐γ, TNF‐α) and expression of the degranulation marker CD107a, further validating their direct interference with NK effector functions [69].

Notably, recent studies have uncovered reverse regulatory effects of NK cells on MDSCs. Activated NK cells can reprogram MDSCs via interaction between their surface NKG2D receptors and NKG2D ligands (NKG2DLs) expressed on MDSCs, converting these cells from an immunosuppressive to a pro‐inflammatory phenotype characterized by secretion of effector molecules such as TNF‐α and IFN‐γ [70, 71]. Building upon this, exogenous administration of the NKG2D‐Fc fusion protein (comprising the extracellular domain of NKG2D and the immunoglobulin Fc segment) selectively eliminates MDSCs by targeting NKG2DLs on their surface via antibody‐dependent cellular cytotoxicity (ADCC). This strategy enhanced NK cell proportions and amplified anti‐tumor immune responses in murine tumor models [72]. These findings provide critical theoretical foundations for developing novel immunotherapies targeting the NK‐MDSC axis.

#### 3.4.3. B‐Cells

The interaction between MDSCs and B lymphocytes plays a pivotal regulatory role in immune‐related diseases. Human PMN‐MDSCs suppress B‐cell proliferation and IgM secretion via a contact‐independent mechanism involving upregulated IDO, Arg‐1, and nitric oxide (NO) [73], suggesting MDSCs may constitutively restrain autoreactive B‐cell clones under homeostasis. In multiple sclerosis studies, the frequency of CD138^+^ B‐cells in patient cerebrospinal fluid inversely correlates with PMN‐MDSC levels. Experimental autoimmune encephalomyelitis (EAE) models further revealed that conditional knockout of *STAT3* in Ly6G^+^ cells depletes PMN‐MDSCs, leading to B‐cell accumulation in the central nervous system (CNS). This accumulation drives microglial polarization toward a pro‐inflammatory phenotype, exacerbating demyelination and impairing neurological recovery [74]. In SLE, loss of M‐MDSCs promotes B‐cell hyperproliferation and activation by suppressing the mesenchymal‐epithelial transition factor protein (Met)/COX2/PGE2 signaling axis. This process positively correlates with SLE disease activity [75]. Conversely, within the TME, breast tumor‐derived MDSCs activate the PD‐L1/PI3K/AKT/NF‐κB signaling cascade, inducing the generation of immunosuppressive PD‐1‐PD‐L1^+^ regulatory B‐cells (Bregs) [76, 77]. Collectively, these studies underscore that MDSCs dynamically regulate B‐cell functions through microenvironment‐specific molecular pathways, thereby exerting immunomodulatory effects in both autoimmune disorders and cancer. These findings highlight that MDSCs dynamically regulate B‐cell function through disease‐specific pathways, with their role in B‐cell‐driven conditions like ITP requiring further investigation.

#### 3.4.4. Dendritic Cells

Dendritic cells (DCs), as pivotal antigen‐presenting cells (APCs), play a critical role in the TME. Studies have revealed that bone marrow MDSCs under co‐stimulation with lipopolysaccharide (LPS) and IFN‐γ not only markedly inhibit the development and maturation of DCs but also enhance their immunosuppressive capacity by augmenting NO secretion [78]. Furthermore, PMN‐MDSCs impair DC antigen presentation by transferring oxidatively truncated lipids to them [79]. It remains a plausible hypothesis that in the immune attack of AA, the loss of MDSCs may indirectly enhance the antigen‐presenting capacity of DCs, thereby amplifying the assault of T cells on HSCs [80]. Given the central role, DC‐based vaccines have emerged as a key strategy in cancer immunotherapy, showing efficacy alone or in combination with immunomodulatory drugs (iMiDs) or chemotherapeutic agents [81–84]. However, in autoimmune contexts such as SLE models, MDSCs were found to exacerbate autoimmune responses by activating the Toll‐like receptor 7 (TLR7) signaling pathway in DCs [85]. Therefore, rebalancing the interplay between MDSCs and DCs is essential for therapeutic optimization.

### 3.5. Context‐Dependent Functional Plasticity: Pro‐Inflammatory Effects and Transcriptional Reprogramming of MDSCs

**Figure 2:**
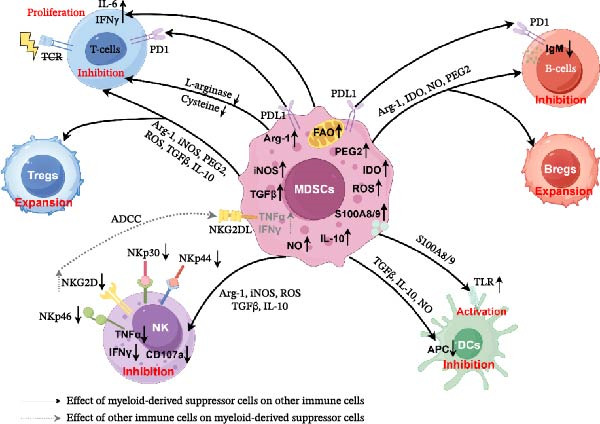
Schematic representation of MDSC‐mediated immune suppression, pro‐inflammatory effect, and cross‐talk with adaptive and innate immune subsets. MDSCs use various mechanisms to promote expansion of Tregs and Bregs and suppress the function of T cells, NK cells, and DCs. Under specific conditions, MDSCs exhibit pro‐inflammatory effects by promoting T‐cell proliferation and DCs activation. This figure was originally created by Figdraw (www.figdraw.com).

It is increasingly recognized that the gene expression profiles and differentiation trajectories of MDSCs exhibit marked disease specificity, which in turn governs their terminal functional commitment across various pathological conditions. In patients with myocardial infarction, immature CD14^+^HLA‐DR^neg/low^ monocytes lose their ability to suppress T‐cell proliferation and form a synergistic pro‐inflammatory axis with expanded peripheral CD16^+^CD66b^+^CD10^neg^ neutrophils. These cells collectively drive excessive inflammatory responses through direct cell–cell contact or inflammatory cytokine cross‐talk [86]. In both chronic graft‐versus‐host disease (cGVHD) and SLE, the frequency of LDNs is significantly elevated, predominantly exhibiting an immature CD10‐negative phenotype. These LDNs directly contribute to pathological amplification of autoimmune responses by enhancing T‐cell proliferation and promoting IL‐6 and IFN‐γ secretion [87, 88]. Notably, the generation of pro‐inflammatory LDNs may share cross‐disease mechanisms: even in healthy donors, granulocyte G‐CSF stimulation induces phenotypically similar immature CD66b^+^CD10^−^ LDNs, which similarly participate in immune activation by augmenting T‐cell activity and IFN‐γ production [88] (Figure 2). In the psoriatic microenvironment, MDSCs lose their suppressive capacity and instead promote IL‐17 production via the RORγt/NFAT1 axis, thereby exerting pro‐inflammatory effects [17]. In rheumatoid arthritis (RA), M‐MDSCs drive abnormal osteoclast differentiation and bone erosion by establishing a positive feedback loop with Th17 cells [89]. In secondary progressive multiple sclerosis (SPMS), M‐MDSCs not only lose their inhibitory function but also promote T‐cell proliferation, consistent with a pro‐inflammatory phenotype that may be associated with reduced IL‐10 and heme‐oxygenase‐1 (HMOX‐1) [90]. Therefore, the mere accumulation of MDSCs is insufficient to predict their pathological impact; their ultimate functional role is determined by differentiation and transcriptional reprogramming programs directed by the local microenvironment.

## 4. The Role of MDSCs in Immune‐Mediated Hematologic Diseases

Most studies have shown that an increase in MDSCs is associated with high tumor burden, advanced tumor stage, poor clinical outcomes in patients, and hematologic malignancies [91–93]. However, MDSCs are not solely detrimental; particularly in immune‐mediated hematologic diseases, they play a positive role in the immune microenvironment. The relevant studies are summarized in Table 1.

**Table 1:** MDSCs in immune‐mediated hematologic diseases.

Disease case	MDSCs phenotype definition	Clinical findings and mechanism	Year/reference
ITP, *n* = 25	CD33^+^CD11b^+^HLA‐DR^-^ MDSCs	Decreased MDSCs vs. healthy controls; circulating MDSCs and Tregs decreased simultaneously in ITP patients; the recovery of MDSCs correlated with treatment response	2017 [94]
ITP, *n* = 80	CD33^+^CD11b^+^HLA‐DR^-^ MDSCs	MDSCs were predictors for ITP initial (6 days) and prolonged (6 months) treatment response	2021 [95]
ITP, *n* = 62	CD33^+^CD11b^+^HLA‐DR^-^ MDSCs	TPO‐RA treatment enhanced MDSC proportion and function in ITP patients, potentially by downregulating KLF9	2024 [96]
ITP, *n* = 21	CD33^+^CD11b^+^HLA‐DR^low^ MDSCs	MDSCs in ITP patients were deficient in both number and immunosuppressive function in the PB and spleen (a) defect that was restored by HD‐DXM treatment through the upregulation of Ets1	2016 [97]
ITP, *n* = 33	CD11b^+^CD33^+^HLA‐DR^-^ MDSCs	The percentages of M2‐like macrophages and MDSCs were both increased significantly in CR group, potentially associated with MIP‐1α/CCL3, MCP‐1, eotaxin‐1/CCL11 and IL‐1β expression	2018 [98]
ITP, *n* = 36	CD11b^+^CD33^+^HLA‐DR^-^ MDSCs	Glucocorticoid resistance in ITP stems from a GR deficiency in MDSCs, which impairs mitochondrial metabolism and fatty acid oxidation, thereby diminishing immunosuppressive function	2022 [99]
ITP, *n* = 43	CD11b^+^CD33^+^HLA‐DR^-^ MDSCs	Low‐dose decitabine upregulates LKB1 to enhance the metabolic and immunosuppressive functions of MDSCs, thereby ameliorating ITP	2022 [100]
ITP, *n* = 72	CD33^+^CD11b^+^HLA‐DR^low^ MDSCs	Platelet‐derived TGF‐β1 mediates TPO‐RA‐induced expansion and functional reprogramming of MDSCs via the TGF‐β/Smad pathway, promoting immune homeostasis in ITP	2024 [101]
AA, *n* = 65	CD11b^+^CD33^+^HLA‐DR^-^ MDSCs, CD33^+^CD11b^+^HLA‐DR^-^CD15^+^CD14^-^ G‐MDSCs, CD33^+^CD11b^+^HLA‐DR^-^CD15^-^CD14^+^ M‐MDSCs	Decreased number and immunosuppressive functions of MDSCs in AA patients compared to healthy controls, which could be rescued by rapamycin	2022 [102]
AA, *n* = 27	CD11b^+^CD33^+^HLA‐DR^-^ MDSCs	In acquired AA, MDSC levels decrease but recover following treatment. In contrast, congenital AA is characterized by a pronounced increase in MDSCs compared to healthy controls	2024 [103]
AA animals	CD11b^+^Ly6G^+^Ly6C^low^ G‐MDSCs	G‐MDSCs attenuate immune‐mediated marrow failure in a minor histocompatibility antigen‐mismatched murine model by inhibiting T‐cell responses and preserving hematopoietic cells.	2023 [104]
AA animals	CD11b^+^Ly6G^+^Ly6C^low^ G‐MDSCs	G‐MDSC‐exosomes attenuate bone marrow failure via modulating the delivery of immunosuppressive miRNAs into activated T lymphocytes	2023 [105]
GVHD, *n* = 5	CD33^+^CD11b^+^HLA‐DR^-^CD15^-^CD14^+^ M‐MDSCs	M‐MDSCs percentages are increased in patients treated with PTCy, which augment Tregs recovery.	2023 [61]
GVHD animals	CD11b^+^Ly6G^+^Ly6C^low^ G‐MDSCs	Cyclosporine A inhibits MPTP opening to restore the immunosuppressive function of G‐MDSCs impaired in GVHD	2022 [62]
haplo‐HSCT for SCD, *n* = 20	lin^-^HLA‐DR^-^CD11b^+^CD33^+^ e‐MDSCs	Elevated frequencies of e‐MDSCs at post‐transplant Day 30 were observed in engrafted patients and those with high donor myeloid chimerism, and this increase was correlated with Treg levels at Day 100	2021 [106]
GVHD, *n* = 76	HLA‐DR^−^/^low^CD33^+^CD16^−^ e‐MDSCs	Negative correlations between the frequency of e‐MDSCs among donor PBSCs and the development of aGVHD in recipients	2019 [107]
GVHD animals	CD11b^+^Gr‐1^+^ MDSCs	hUCMSCs reduced the incidence and severity of GVHD by enriching MDSCs in target tissues	2021 [108]
CIN, *n* = 102	CD33^+^CD11b^+^HLA‐DR^-/low^CD15^+^ G‐MDSCs, CD33^+^CD11b^+^HLA‐DR^-/low^ CD14^+^ M‐MDSCs	Decreased MDSCs vs. healthy controls; Transcriptomics revealed altered MDSC function in CIN, with upregulation of pro‐inflammatory, T‐cell activation/response pathways and downregulation of DNA damage repair and phagocytosis.	2024 [109]

Abbreviations: AA, aplastic anemia; CIN, chronic idiopathic neutropenia; e‐MDSCs, early MDSCs; G‐MDSCs, granulocytic MDSCs; GR, glucocorticoid receptor; GVHD, graft‐versus‐host disease; haplo‐HSCT, haploidentical hematopoietic stem cell transplantation; HD‐DXM, high‐dose dexamethasone; hUCMSCs, human umbilical cord‐derived mesenchymal stem cells; ITP, immune thrombocytopenia; M‐MDSCs, monocytic MDSCs; MDSC, myeloid‐derived suppressor cells; MPTP, mitochondrial permeability transition pore; PB, peripheral blood; PBSCs, peripheral blood stem cells; PTCy, posttransplantation cyclophosphamide; SCD, sickle cell disease; TPO‐RA, TPO receptor agonists.

### 4.1. ITP

ITP, an autoimmune disorder characterized by antibody‐mediated platelet destruction and impaired platelet production, is closely associated with defects in immunosuppressive cellular networks [110]. Studies demonstrate that ITP patients exhibit significantly reduced proportions of MDSCs and Tregs in PB compared to healthy individuals, with further depletion of MDSCs observed in relapsed cases [94]. Dynamic changes in MDSC levels not only correlate with disease activity but are also positively associated with initial therapeutic response rates and sustained remission, suggesting MDSCs as potential biomarkers for assessing immune homeostasis and treatment prognosis in ITP [95, 96]. Notably, ITP patients demonstrated impaired suppressive function and number of MDSCs, which could be rescued by high‐dose dexamethasone (HD‐DXM) treatment in responders [97, 98]. MDSCs from HD‐DXM nonresponders exhibit mitochondrial metabolic abnormalities, including downregulation of carnitine palmitoyltransferase‐1 (CPT‐1), a key enzyme in FAO, and glucocorticoid receptor (GR). Animal models confirm that GR‐deficient MDSCs lose their capacity to ameliorate thrombocytopenia, implicating dysregulation of the metabolic‐immune axis as a central mechanism underlying glucocorticoid resistance [99]. Conversely, low‐dose decitabine enhances MDSCs generation and augments their aerobic metabolism and immunosuppressive function by upregulating liver kinase B1 (LKB1) expression [100]. Additionally, sustained remission following thrombopoietin receptor agonist (TPO‐RA) discontinuation may involve platelet‐derived TGF‐β1, which amplifies MDSCs proliferation and immunosuppressive activity via the TGF‐β1‐Smad2/3 pathway, revealing a novel bidirectional platelet‐immune cell regulatory mechanism [101].

### 4.2. AA

AA, an immune‐mediated bone marrow failure syndrome marked by pancytopenia and hypoplastic marrow, is driven by aberrant CTL activation and Treg dysfunction [111]. Recent studies reveal dynamic imbalances in MDSCs and their subsets during AA pathogenesis: Dong et al. [102] reported significantly impaired numbers and immunosuppressive functions of MDSCs in acquired AA patients, particularly M‐MDSCs, whose reduction correlates positively with peripheral Treg frequency, bone marrow *WT1* expression, and plasma Arg‐1 levels. Intriguingly, congenital AA patients exhibit markedly higher proportions of MDSCs and their subsets (G‐MDSCs, M‐MDSCs, and e‐MDSCs) compared to acquired AA, suggesting MDSCs as potential biomarkers for differential diagnosis [103]. Furthermore, Young’s team first demonstrated that G‐MDSCs and their secreted exosomes ameliorate pancytopenia and suppress T‐cell bone marrow infiltration in major histocompatibility complex (MHC)‐matched AA murine models. However, this protective effect diminishes in MHC‐mismatched models due to altered immune microenvironments, indicating that MDSC‐mediated immunoregulation depends on host‐donor immune compatibility [104, 105]. In addition, further clinical evidence supports the role of myeloid‐derived cells in AA and highlights that macrophages, correlating with defective MDSC function, contribute to disease pathogenesis and progression [112].

### 4.3. GVHD

Allogeneic hematopoietic stem cell transplantation (allo‐HSCT), a curative therapy for high‐risk/refractory hematologic malignancies and non‐malignant disorders [113], is limited by GVHD, a process where donor‐derived alloreactive T cells recognize host antigens, infiltrate target organs (e.g., skin, gut, liver), and trigger inflammatory cascades [114]. PTCy, a cornerstone for acute GVHD prophylaxis administered on days +3 to +4, induces dysfunction of alloreactive T cells [115]. Fletcher et al. [61] found that PTCy significantly increases peripheral G‐MDSCs and M‐MDSCs frequencies in both murine models and allo‐HSCT patients, while indirectly modulating Treg expansion. Mitochondrial permeability transition pore (MPTP) hyperactivation in acute GVHD microenvironments causes mitochondrial damage and functional loss in donor PMN‐MDSCs. CsA restores their immunosuppressive capacity by inhibiting MPTP activation, uncovering a novel mechanism of CsA‐mediated MDSCs regulation in GVHD [62]. Clinical cohort data reveal that expanded e‐MDSCs correlate positively with donor myeloid chimerism after haploidentical transplantation in sickle cell disease [106]. Additionally, an increased level of MDSCs in the grafts could mitigate acute GVHD [116]. Specifically, higher numbers of HLA‐DR^-/low^CD33^+^CD16^-^ MDSCs in donor grafts are protective against grade II–IV acute GVHD in recipients [107].

Given the beneficial effects of MDSCs in GVHD therapy, their mechanisms of action and corresponding interventions have been actively investigated. For instance, in murine GVHD models, the exogenous administration of IL‐27 or GM‐CSF to promote the expansion of MDSCs has been shown to reduce disease incidence [117, 118]. Human umbilical cord mesenchymal stem cells (UC‐MSCs) promote MDSCs homing to GVHD target organs via CXCL1 secretion, creating an immunoprotective niche and providing a rationale for MDSC‐targeted therapeutic strategies [108]. Inflammation is a critical driver of GVHD pathogenesis. Under such conditions, the loss of myeloid IDO1 impairs ROS scavenging, promoting the differentiation of MDSCs into pro‐inflammatory neutrophils and compromising their immunosuppressive function [119]. To address this limitation, the team led by Bruce R. Blazar generated inflammasome‐resistant MDSCs from induced pluripotent stem cells (iPSCs), which retained up to 95% of their immunosuppressive capacity [120].

### 4.4. CIN

CIN is characterized by the persistent and unexplained reduction of PB absolute neutrophil counts. A central mechanism in the pathophysiology of CIN is a pro‐apoptotic bone marrow microenvironment, which promotes the destruction of granulocyte progenitors and is mediated by aberrant immune cells [121, 122] In CIN, MDSCs are present at significantly reduced numbers and exhibit a distinct transcriptomic profile indicative of both heightened inflammatory activity and impaired cellular viability, suggesting their involvement in the disease’s pathophysiology [109].

## 5. The Role of MDSCs in Hematological malignancies

MDSCs play a crucial immunosuppressive and tumor‐promoting role across hematological malignancies. In lymphoma, elevated MDSCs levels, particularly M‐MDSCs, correlate with multi‐drug resistance and poor prognosis [123]. Their expansion is regulated by factors such as PD‐L1 and IL‐35 and is linked to vitamin D deficiency, while targeting MDSCs can enhance treatment response [124–126]. In multiple myeloma, MDSCs accumulate extensively in the bone marrow microenvironment, where they interact with tumor cells and mesenchymal stromal cells via exosomes, S100A9, galectin‐1, and other mediators, collectively fostering immune evasion, bone lesion progression, and therapy resistance [127, 128]. Interventions such as anti‐CD38 antibodies or demethylating agents can reduce MDSCs numbers [81, 129]. In leukemia, higher MDSCs levels in acute leukemia are associated with minimal residual disease and inferior outcomes [130, 131]. In chronic myeloid leukemia and chronic lymphocytic leukemia, MDSCs expansion sustains immunosuppression, whereas tyrosine kinase inhibitors (TKIs, e.g., imatinib, dasatinib) and Bruton’s tyrosine kinase inhibitors (e.g., ibrutinib) effectively lower MDSCs frequencies and improve the immune microenvironment [132–134]. In myelodysplastic syndromes, MDSCs suppress normal hematopoiesis and drive CD8^+^ T‐cell exhaustion through mechanisms including S100A9 secretion and overexpression of PD‐L1 and galectin‐9, thereby promoting clonal expansion and disease progression [135, 136]. In summary, MDSCs act as central regulators of the immunosuppressive TME in various blood cancers, and targeting them or their associated pathways represents a promising therapeutic strategy to improve clinical outcomes.

## 6. Future Perspective

Although preliminary progress has been made in understanding the role of MDSCs in immune‐mediated hematologic diseases, their complex functional plasticity and microenvironmental dependency continue to pose challenges for mechanistic elucidation and clinical translation. Future research must delve into the following dimensions.

First is the resolution of MDSC heterogeneity and precision‐targeted regulation. Current definitions of MDSCs still rely on a combination of phenotypic markers and functional validation, but the functional differentiation and molecular characteristics of their subsets (e.g., G‐MDSCs, M‐MDSCs, and e‐MDSCs) across different diseases remain incompletely elucidated. Future studies should employ single‐cell transcriptomics and epigenetic technologies to unveil subset‐specific regulatory networks or design targeted depletion/functional reprogramming strategies based on MDSCs surface markers (e.g., LOX‐1, FATP2) or metabolic features (e.g., lipid uptake dependency, FAO), thereby providing a molecular foundation for developing MDSC‐directed interventions.

Second is the functional integration of metabolic‐immune regulatory axes. The immunosuppressive functions of MDSCs are highly dependent on metabolic reprogramming (e.g., amino acid depletion via Arg‐1/iNOS, FAO‐driven energy supply), yet the spatiotemporal dynamics of their metabolic networks and their association with disease progression require further exploration. For example, is MDSCs functional impairment in AA directly linked to mitochondrial metabolic abnormalities (e.g., excessive MPTP opening)? Can the molecular mechanism by which decitabine enhances aerobic metabolism in MDSCs from ITP be extrapolated to other immune‐mediated hematologic disorders? Integrating metabolomics with functional gene‐editing technologies to dissect regulatory nodes of key metabolic enzymes or signaling pathways may identify novel targets to reverse immune dysregulation.

Third is cross‐disease mechanistic comparisons and identification of universal principles. The functional divergence of MDSCs in cancer, autoimmune diseases, and transplantation‐related pathologies suggests microenvironment‐specific regulation, yet certain mechanisms may exhibit cross‐disease. For instance, do metabolic abnormalities in MDSCs from the TME and AA bone marrow failure share common signaling hubs (e.g., STAT3 or FAO pathways)? Comparative analysis of MDSCs interactions with immune cells (e.g., Tregs, Bregs, and NK cells) across pathological contexts may reveal universal regulatory principles, offering theoretical support for developing broad‐spectrum immunomodulators. Additionally, establishing biomarker systems based on MDSCs dynamics will facilitate personalized therapy and treatment response prediction.

Fourth is the translation of mechanistic insights into evidence‐based therapeutic strategies. A central paradigm in MDSC‐targeted therapy is their context‐dependent utility: in immune‐mediated hematologic diseases, MDSCs exert beneficial immunosuppression by restraining pathogenic T‐ and B‐cells, whereas in malignancies, this same function fosters tumor immune evasion and therapy resistance. Notably, several existing therapies already engage MDSCs biology, providing foundational proof‐of‐concept. In ITP, glucocorticoids, low‐dose decitabine, and TPO receptor agonists have demonstrated efficacy in restoring MDSCs function [96, 99, 100]. In GVHD, PTCy and CsA exert protective effects partly through MDSCs expansion and functional recovery [62, 115]. These observations validate MDSCs as a druggable immunoregulatory node and support the repurposing of approved agents. Therapeutic strategies targeting tumor‐associated MDSCs are designed to intervene through multiple mechanisms, including promoting their differentiation into mature myeloid cells (e.g., all‐trans retinoic acid, STAT3 inhibitor, Toll‐like receptors), inhibiting their proliferation and recruitment (e.g., anti‐chemokine/chemokine receptors, anti‐GM‐CSF/G‐CSF, anti‐VEGF/VEGFR, CD33 inhibitor), suppressing their immunosuppressive functions (e.g., phosphodiesterase 5 inhibitors, activators of the nuclear factor erythroid 2‐related factor 2, histone deacetylase inhibitors), depleting existing MDSCs populations (e.g., chemotherapy), and disrupting their metabolic pathways [92, 137]. Future efforts should aim to tailor these approaches to specific disease contexts—enhancing MDSC‐mediated suppression in autoimmune settings while effectively inhibiting or reprogramming MDSCs in cancer—guided by robust biomarkers and validated through rigorous preclinical and clinical studies.

## 7. Conclusions

In summary, MDSCs research is transitioning from phenotypic description to mechanistic depth and clinical translation. Through multi‐omics integration, innovations in dynamic monitoring technologies, and interdisciplinary collaboration, breakthroughs in precision immunotherapy targeting MDSCs regulation may be achieved for immune‐mediated hematologic diseases.

## Author Contributions

Qi Liu, Kunpeng Zhang, Shun He, and Xinru Jia wrote the paper. Jingjing Liu, Dijiong Wu, and Baodong Ye examined and modified the paper.

## Funding

The present study was supported by the National Natural Science Foundation of China (Grants 82274273, and 82174138) and the Specific Program of Scientific Research of Zhejiang Chinese Medicine University for Affiliated Hospital (Grant 2025FSYYZX04).

## Disclosure

All authors assisted in the manuscript and were responsible for the work.

## Conflicts of Interest

The authors declare no conflicts of interest.

## Data Availability

Data sharing is not applicable to this article as no datasets were generated or analyzed during the current study.
